# Facing the Challenges of Neuropeptide Gene Knockouts: Why Do They Not Inhibit Reproduction in Adult Teleost Fish?

**DOI:** 10.3389/fnins.2018.00302

**Published:** 2018-05-03

**Authors:** Vance L. Trudeau

**Affiliations:** Department of Biology, University of Ottawa, Ottawa, ON, Canada

**Keywords:** neuropeptides, neurotransmitters, luteinizing hormone, GnRH, kisspeptins, transgenic, knockout, reproduction

## Abstract

Genetic manipulation of teleost endocrine systems started with transgenic overexpression of pituitary growth hormone. Such strategies enhance growth and reduce fertility, but the fish still breed. Genome editing using transcription activator-like effector nuclease in zebrafish and medaka has established the role of follicle stimulating hormone for gonadal development and luteinizing hormone for ovulation. Attempts to genetically manipulate the hypophysiotropic neuropeptidergic systems have been less successful. Overexpression of a gonadotropin-releasing hormone (*gnrh*) antisense in common carp delays puberty but does not block reproduction. Knockout of Gnrh in zebrafish does not impact either sex, while in medaka this blocks ovulation in females without affecting males. Spawning success is not reduced by knockout of the kisspeptins and receptors, agouti-related protein, agouti signaling peptide or spexin. Hypotheses for the lack of effect of these genome edits are presented. Over evolutionary time, teleosts have lost the median eminence typical of mammals. There is consequently direct innervation of gonadotrophs, with the possibility of independent regulation by >20 neurohormones. Removal of a few may have minimal impact. Neuropeptide knockout could leave co-expressed stimulators of gonadotropins functionally intact. Genetic compensation in response to loss of protein function may maintain sufficient reproduction. The species differences in hypothalamo-hypophysial anatomy could be an example of compensation over the evolutionary timescale as teleosts diversified and adapted to new ecological niches. The key neuropeptidergic systems controlling teleost reproduction remain to be uncovered. Classical neurotransmitters are also regulators of luteinizing hormone release, but have yet to be targeted by genome editing. Their essentiality for reproduction should also be explored.

## Introduction

The first transgenic teleosts were goldfish engineered to express human growth hormone (Gh^1^; Zhu et al., 1985). Overexpression of Gh disrupts reproduction in fishes, but it does not inhibit it completely. For example, some male Gh-transgenic tilapia have lower levels of sperm production while the females have significantly lower ovarian size (Rahman et al., 1998, 2001). Common carp overexpressing Gh have enhanced growth but have delayed gonadal development (Cao et al., 2014). Coho salmon transgenic for Gh also have reduced reproductive success, but they can spawn with wild fish (Leggatt et al., 2014). This presents a challenge for the aquaculture industry and environmental regulators concerned with the potential effects of genetically-modified organisms on native populations. The search for strategies to produce fast-growing fish that do not reproduce remains a significant challenge.

Attempts to manipulate follicle stimulating hormone (Fsh) and luteinizing hormone (Lh) are yielding interesting results. Wei Ge's group has used transcription activator-like effector nuclease (TALEN) to delete gonadotropin β subunits. Both female and male *fshb-*deficient zebrafish were fertile, but both ovarian and testicular development were delayed. In contrast, *lhb-*deficient zebrafish showed normal gonadal growth, but the females failed to spawn. By analysis of sex ratios and the histological presence of intersex individuals, this group also showed that Fsh may play a role in maintaining female status, probably through regulation of ovarian aromatase (Zhang et al., 2015b). Both supportive and contradictory evidence was obtained when they disrupted gonadotropin receptor expression (Zhang et al., 2015a). Neither Fshr nor Lhcgr deficiency mimicked deficiencies of their ligands. This is likely due to the fact that zebrafish Fshr can be activated by both the Fsh and Lh proteins. They found that Fshr was indispensable to folliculogenesis and the disruption of the *fshr* gene resulted in a complete failure of follicle activation, followed by masculinization of females into males. In contrast, Lhcgr does not seem to be essential to zebrafish reproduction in both sexes. It has long been known that teleost Fsh (formerly called gonadotropin-I) and Lh (gonadotropin-II) are more similar to each other than are the 2 tetrapod gonadotropins (Levavi-Sivan et al., 2010). TALENs have been employed to disrupt gonadotropin β subunit expression in medaka (Takahashi et al., 2016). Female homozygotes for *fshb* and *lhb* null fish were infertile but males were fertile. They established that Fshb is required for gonadal development, whereas Lhb is required for final gonadal maturation and is essential for ovulation in medaka.

Such successes with pituitary hormones have not been mirrored in the attempts to manipulate the neuropeptidergic systems. Here, I present key developments and several hypotheses about why neuropeptide knockouts have thus far not completely inhibited reproduction in adult teleosts.

## Knockout of Gnrh does not inhibit reproduction

Following the isolation of neuropeptides in the 1970s, it was quickly established that exogenous mammalian Gnrh could enhance Lh secretion in fishes (Breton and Weil, 1973; Crim and Cluett, 1974). Now we know that 3 distinct isoforms of Gnrh usually co-exist in the modern teleosts (e.g., perch-like fishes). There is the species-specific hypophysiotropic Gnrh1 in the pre-optic area and regions of the hypothalamus, the near ubiquitous (except for rodents) Gnrh2 in the midbrain, and Gnrh3 in the terminal nerve and ventral telencephalon (Powell et al., 1994; Coe et al., 1995; Steven et al., 2003; Spicer et al., 2016). In salmon, goldfish and zebrafish, only Gnrh2 and Gnrh3 are present. In these fish, it appears that Gnrh3 has taken over the role of Gnrh1 to stimulate Lh release (Tello et al., 2008; Roch et al., 2014). Supporting this are data in Atlantic cod, where *gnrh1* is a pseudogene, *gnrh2* is expressed, and *gnrh3* is the hypophysiotropic form (Hildahl et al., 2011).

The multiplicity and complexity of the neuroendocrine control of teleost reproduction has long been recognized (Peter et al., 1990; Trudeau, 1997; Zohar et al., 2010). This includes the Gnrhs, many others neuropeptides, and aminergic and amino acid neurotransmitters (Table 1). Early attempts at genetic manipulation focused on *gnrh* antisense transgenes. In rainbow trout expressing *gnrh*-antisense RNA under the control of the Atlantic salmon *gnrh3* promoter (Uzbekova et al., 2000), there were no major effects on the levels of circulating Fsh and Lh or time of maturation. Subsequent studies have revealed a more important role for Gnrh3 in common carp (Xu et al., 2011). Abnormal sexual development and infertility were observed in ~40% of the carp expressing the *gnrh3*-antisense transgene. The remaining 60% had normal gonads with sperm or eggs, indicating that reproduction was still possible. Part of the reason for partially disrupted reproduction lies in the effects of the antisense *gnrh* transgene to reduce expression of gonadotropin common α *cga* and *fshb* subunits in the pituitary. On the other hand*, lhb* mRNA levels in the pituitary and serum levels of Lh were not affected, which may explain why some carp develop and breed normally (Xu et al., 2011).

**Table 1:** Stimulatory and inhibitory neuropeptides, aminergic and amino acid neurotransmitters shown to regulate luteinizing hormone (Lh) in various teleost species.

**Neuroendocrine regulators of Lh in teleosts**	Associated genes^*^	**Key citation**
**1. STIMULATORY NEUROPEPTIDES**		
(a) agouti-related peptide	*agrp*	Zhang et al., 2012
(b) cholecystokinin 8S	*ccka, cckb*	Trudeau, 1997
(c) galanin	*galn*	Pinto et al., 2017
(d) gonadotropin-releasing hormone	*gnrh1, gnrh2, gnrh3*	Zohar et al., 2010
(e) isotocin	*oxt*	Popesku et al., 2008
(f) kisspeptin	*kiss1, kiss2*	Li et al., 2009
(g) melanocortin	*pomca, pomcb*	Jiang et al., 2017
(h) neurokinin B	*tac3a, tac3b*	Biran et al., 2012
(i) neuropeptide Y	*npy*	Zohar et al., 2010
(k) pituitary adenylate cyclase activating peptide	*adcyap1a, adcyap1b*	Chang et al., 2009
(k) secretoneurin	s*cg2a, scg2b*	Trudeau et al., 2012
**2. INHIBITORY NEUROPEPTIDES**		
(a) ghrelin	*ghrl*	Chang et al., 2009
(b) growth hormone-releasing hormone	*ghrh*	Grey and Chang, 2013
(c) gonadotropin-release inhibiting hormone (− and +)	*npfv*	Muñoz-Cueto et al., 2017
(d) spexin	*spx*	Zheng et al., 2017
**3. AMINES**		
(a) dopamine (−)	*th1, th2*	Popesku et al., 2008
(b) norepinephrine (+)	*dbh*	Trudeau, 1997
(c) serotonin (+)	*tph1a, tph1b*	Popesku et al., 2008
**4. AMINO ACIDS**		
(a) γ-aminobutyric acid (+)	*gad1, gad2, gad3*	Trudeau et al., 2000
(b) glutamate (+)	*gsla, gslb*	Trudeau et al., 2000
(c) taurine (+)	*csad, gadl1*	Trudeau, 1997

*The + and − indicate stimulation and inhibition, respectively. Also shown are the principle genes involved in the synthesis of the listed regulatory factors. Key references are given, acknowledging that there are many other excellent papers that have not been cited due to space limitations*.

* *Gene names are taken from http://useast.ensembl.org/Danio_rerio/Info/Index*.

Destruction of Gnrh3 neurons by laser ablation early in development (4–6 days post-fertilization; dpf) negatively impacts reproduction in adult zebrafish (Abraham et al., 2010). Mature animals lacking Gnrh3 neurons had arrested oocyte development and reduced average oocyte diameter. Those animals with confirmed total ablation of Gnrh3 neurons failed to ovulate. It was noted that when ablation of Gnrh3 cells was conducted at 2 dpf, high Gnrh3 neuronal regeneration rates were observed, but this regeneration capacity significantly decreased when ablation was performed at 4 or 6 dpf (Abraham et al., 2010). The same group showed that transient Gnrh3 gene knockdown using anti-sense morpholino oligonucleotides resulted in misguided migration of Gnrh3 neurons during neurogenesis (Abraham et al., 2008). Therefore, the development and migration of the Gnrh3 neuronal system is essential to its proper functioning in the adult zebrafish. In this sense, one can conclude that the hypophysiotropic Gnrh plays an important role in zebrafish, mirroring some aspects of the situation in mammals (e.g., Kallman's syndrome in humans; Soussi-Yanicostas et al., 1998).

Zohar's group subsequently targeted zebrafish Gnrh3 neurons for mutagenesis using TALEN methodologies (Spicer et al., 2016). Surprisingly, they observed no effects on reproduction. Early dynamic alterations in the expression of *fshb, lhb* and *cga* in the developing mutants returned to normal in the adults. The authors postulated that a compensatory mechanism must exist to account for the lack of effect of the Gnrh3 knockout. This could involve upregulation of the Gnrh2 system, yet even the Gnrh3/Gnrh2 double knockout animals were fertile (Spicer et al., 2016). These observations challenge the notion that Gnrh is essential to reproduction. It is clear however, that the method of elimination of Gnrh has a major impact on the results- ranging from infertility in the laser ablation studies to no observable effects in the knockout zebrafish.

Mutation of the hypophysiotropic Gnrh1 using TALEN in medaka revealed an important role in ovulation (Takahashi et al., 2016). Females had well-developed ovaries but failed to ovulate. Ovulation could be partially rescued by injection of Lh. This can be partly explained by the effects of Gnrh1 knockout on gonadotropin gene expression. In females, mutation of *gnrh1* did not affect *fshb*, but had a minor suppressive effect on *lhb* so gonadal development is maintained. It is puzzling that Gnrh1 knockout males remain fertile and no effects on gonadotropin gene expression were observed (Takahashi et al., 2016).

## Kisspeptinergic systems are not essential in small model teleost species

**Figure 1:**
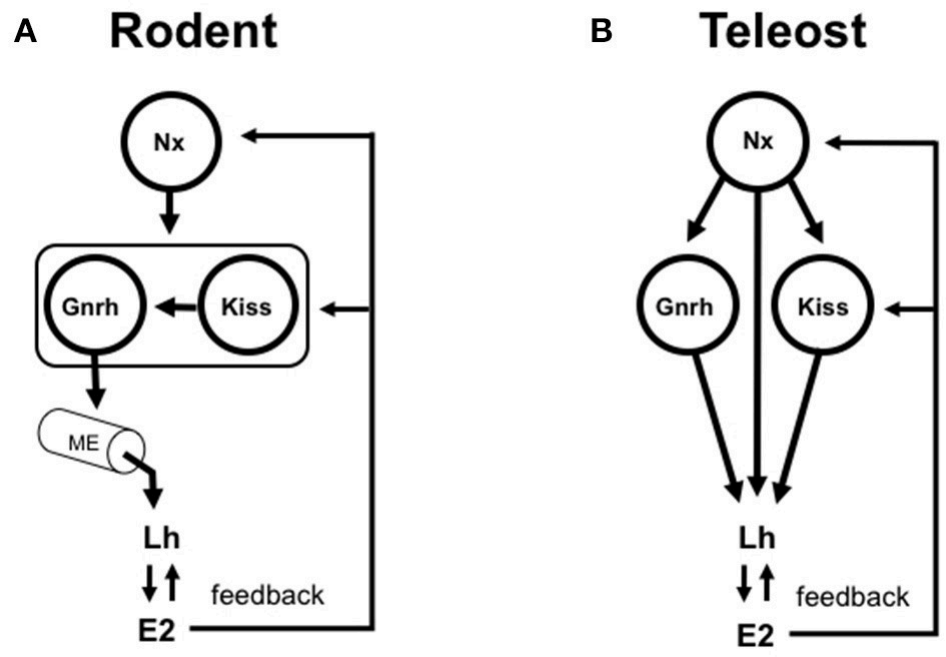
Simplified models depicting the roles of gonadotropin-release hormone (Gnrh) and the kisspeptins (Kiss) in the control of vertebrate reproduction. **(A)** In rodent model species, the Gnrh neuronal system is typically viewed as the key integrator of multiple stimulatory and inhibitory inputs that control Gnrh pulsatility, Lh synthesis and the Lh surge in females. Gnrh neurons project to and release the Gnrh peptide into the median eminence (ME). The hypothalamo-hypophysial portal blood transports Gnrh (and other neuropeptides and neurotransmitters) to the anterior pituitary where gonadotrophs are randomly distributed. Specific populations of Kiss-expressing neurons control specific populations of Gnrh neurons. The Kiss-Gnrh pathway is shown as a functional unit framed with a box. In this context, the Kiss and Gnrh systems are in series. Numerous other neuropeptides and neurotransmitters (Nx; for simplicity, depicted as a single input) are important for the control of Gnrh, kisspeptin and, ultimately Lh release and gonadal function. Gonadal steroids such as estradiol-17β (E2) exert both positive and negative feedback at the levels of hypothalamus and pituitary. Elimination of either Gnrh or Kiss blocks reproduction in a mammal. Experimental data have delineated this essentiality of Gnrh and Kiss in the control of Lh release in mammalian species. **(B)** In teleost fish, the ME has been lost to varying degrees during the course of evolution, and alternatively there is extensive direct innervation of gonadotrophs in the highly regionalized pars distalis of the anterior pituitary. The hypophysiotropic Gnrh neuronal system in teleosts is considered as a key element in the multifactorial direct control of Lh synthesis and release. The role of Kiss is less clear. There is little anatomical evidence supporting the existence of Kiss projections to Gnrh neuronal cell bodies, and Gnrh neurons in teleosts do not express Gpr54. On the other hand, Kiss fibers and Gnrh fibers share a close relationship in the pars distalis. In this context, the Kiss and Gnrh systems (and many others) are in parallel. Gonadal steroids such as E2 exert both positive and negative feedback at the levels of telencephalic preoptic area-hypothalamus and pituitary. Knockout of either Gnrh or Kiss does not block reproduction in a fish. Experimental data have delineated that Gnrh and Kiss are therefore dispensible for reproductive control in zebrafish. Numerous other neurons producing neuropeptides and neurotransmitters (Nx; depicted as multiple inputs. See also Table 1) are important for the control of Gnrh, Kiss and additionally, project to the anterior pituitary for the direct control of Lh and, in turn, gonadal function.

Since the discovery of the link between mutations in the human *gpr54* and isolated hypogonadotropic hypogonadism (de Roux et al., 2003; de Roux, 2006), the kisspeptins have become a key to the central dogma of neuroendocrine regulation of mammalian reproduction (Figure 1). Critical work in several mammalian species has now established that specific kisspeptin neuronal populations directly control Gnrh neurons to regulate Lh release (Clarkson and Herbison, 2009; Fabre-Nys et al., 2017). *In vivo* and *in vitro* studies in fish showed that exogenous kisspeptin peptides could variably enhance Lh release, depending on form and species (Espigares et al., 2015; Zmora et al., 2015). Around the same time he TALEN strategy was employed in zebrafish to knockout kisspeptin 1 and kisspeptin 2 along with their receptors (Tang et al., 2015). Following rigorous analysis, they concluded that the kisspeptin system is dispensable for reproduction. This was surprising, given the close relationship between GnRH3 and kisspeptin nerve fibers in the pars distalis of the zebrafish pituitary (Song et al., 2015). Knockout studies in medaka provides more evidence that kisspeptins are dispensible in teleosts (Nakajo et al., 2018). The same group also show that *in vivo* kisspeptin administration in goldfish does not induce ovulation or increase in serum Lh. This is in clear contrast to earlier work in goldfish showing effects of the kisspeptins on Lh *in vivo* (Li et al., 2009) and from cultured pituitary cells *in vitro* (Chang et al., 2012). Teleost Gnrh neurons lack Gpr54 (Nakajo et al., 2018). These observations are significant for two main reasons: (1) Within the few species studied, there are differences in the importance and action of the kisspeptins; (2) If the kisspeptins have a role, then they must be acting via non-Gnrh systems or directly on gonadotrophs. This implies that the nature of the neuronal circuitry is very different in fishes and is in clear contrast to the kisspeptin-Gnrh link observed in mammalian species so far investigated (Figure 1). Hope for kisspeptin as a reproductive regulator in teleosts is not lost. Some *gpr54-*expressing neurons in key regions (ventral telencephalon and preoptic area) controlling teleost sexual behaviors are the neurons producing neuropeptide b (Npb) (Nakajo et al., 2018). They also show that hypophysiotropic Kiss1-Npb neuronal pathway impinges on isotocin (fish oxytocin) and vasotocin (fish vasopressin) neurons in the preoptic area. This discovery reinforces the idea that the brain circuitry linked to reproduction is likely different between the diverse teleost species.

## Genetic modifications of other neuropeptides do not inhibit reproduction in zebrafish

The importance of agouti-related peptide (Agrp) and melanocortin receptors (mcr4) in the stimulation of larval zebrafish growth has been established (Zhang et al., 2012). The Agrp neurons in the lateral tuberal nucleus are hypophysiotropic and treatment with *agrp* morpholinos in embryos reduced the expression of *gh, fshb* and *lhb* in whole zebrafish larvae at 4 dpf (Zhang et al., 2012). However, the authors did not uncover effects on reproduction in adults of the *mcr4*-/- mutant strain. Overexpression of the melanocortin receptor antagonist agouti signaling peptide (ASIP) had major effects on food intake and growth in zebrafish (Guillot et al., 2016). While no data are presented, the authors state that “*preliminary data support modifications in the puberty timing of ASIP zebrafish.”* Spexin (Spx, neuropeptide Q) suppresses Lh release *in vivo* and *in vitro* in goldfish (Liu et al., 2013). Spexin acts via galanin receptors. While Spx1 knockout increased food intake, it did not affect growth rate in zebrafish (Zheng et al., 2017). The authors concluded that Spx1 may be a satiety signal for feeding control by reducing the expression of orexigenic Agrp1. Regardless, it is clear that none of *agrp, mcr4* or *spx1* modifications prevent breeding in adults, and thus they are not essential for reproduction, at least in zebrafish.

## Reasons why genetic modifications in neuroendocrine systems minimally impact reproduction in teleosts

### Anatomical considerations

Amongst several possibilities, it is important to consider the particular anatomical features of the teleost hypothalamo-hypophysial system. A plausible proposal for the evolution of hypothalamic-pituitary communication has been presented (Ball, 1981; Peter et al., 1990; Gorbman, 1995). Elaborate pituitary vasculature and median eminence-like arrangements appeared early in elasmobranchs (e.g., sharks and rays) ~450 million years ago. The median eminence, typical of tetrapods, is found in numerous classes of the so-called “primitive” fishes, for example, sturgeons, lungfish and coelacanth, with the notable exception being the teleosts. With varying degrees, teleosts have lost the hypothalamo-hypophyseal portal system typical of mammals, and the neurohypophyis has interdigitated with the anterior pituitary. Hypophysiotropic neurons thus terminate in close proximity to the highly regionalized anterior pituitary cells they control (Figure 1). In many cases, there are synapse-like direct contacts with gonadotrophs and somatotrophs in the pars distalis. This relationship is considered derived, and likely arose with the diversification of the teleosts ~250 million years ago. This direct innervation is impressive, with the possibility of independent regulation of gonadotrophs by >20 neuropeptides and neurotransmitters (Peter et al., 1990; Trudeau et al., 2000; Popesku et al., 2008; Zohar et al., 2010). A single gonadotroph *in vitro* can respond to several neuropeptides and neurotransmitters (Chang et al., 2009). Thus, one could alternatively hypothesize that knockout of a single stimulatory or inhibitory neuropeptidergic system would have minimal impact on gonadotropin synthesis and release, and thus reproductive success. Nevertheless, important questions remain- which of the numerous hypophysiotropic factors (Table 1) are essential for reproduction, and which among them are evolutionary conserved?

### Knockout may leave neuronal function intact

It has long been known that certain hypophysiotropic neurons in mammalian model species co-express other neuropeptides or neurotransmitters. This concept has not been well-studied in teleosts, but is likely to exist. All Gnrh neurons in the ovine brain colocalize with galanin (Dufourny et al., 2003). A subpopulation of kisspeptin neurons in the rostral periventricular area expressing dopamine innervate Gnrh neurons in the rostral preoptic area of mice (Clarkson and Herbison, 2011). Double- and triple- labeling experiments have demonstrated that neurons in the arcuate nucleus of the goat co-expressed kisspeptin, neurokinin B, and dynorphin A, and are involved in the control of pulsatile Lh secretion (Wakabayashi et al., 2010). It is thus hypothesized here that knockout of a single neuropeptide in one of these neuronal systems may leave the other co-localized neuropeptide or neurotransmitter intact. Hypothetically, a single gene knockout could turn a stimulatory system (e.g., kisspeptin or Gnrh neurons) into another stimulatory/compensatory neuron (e.g., galanin) or even an inhibitory one (e.g., dopamine). This proposal requires extensive experimental verification in teleosts. In the case of total destruction of a specific stimulatory hypophysiotropic neuronal system would eliminate all neurosecretory material. This would eliminate a key stimulatory pathway, potentially containing multiple activators and result in failed reproduction, as observed with the early developmental elimination of zebrafish Gnrh3 neurons by laser ablation (Abraham et al., 2010).

### Compensatory responses may maintain sufficient reproduction

Knockout and knockdown experiments manipulating *egfl7*, an endothelial extracellular matrix gene, provide direct evidence for activation of a compensatory network to buffer against deleterious mutations in the developing vascular system in the zebrafish (Rossi et al., 2015). Such genetic compensation in response to gene knockout is a widespread phenomenon (El-Brolosy and Stainier, 2017). Upregulation of related genes following a knockout may be a direct consequence of the loss of protein function.

What about compensation in teleost neuroedocrine systems? While conclusive evidence is still lacking, this is a distinct possibility. Knockout of Gnrh3 and the 2 kisspeptin genes in zebrafish undergo normal gonadal maturation (Liu et al., 2017). These fish breed and exhibit normal fertility. Expression of *fshb* and *lhb* in the pituitary was not significantly altered. Expression of neuropeptide Y (*npy*), tachykinin 3 (*tac3*, the neurokinin B encoding gene), and secretogranin-IIa (*scgIIa*) were increased in whole brain extracts in the triple knockout fish. In males, the respective fold increases were 1.3, 1.3, and 1.4 for *npy, tac3* and *scgIIa*. Similarly, in females, the increases were 1.3, 1.2, 2.7-fold for *npy, tac3* and *scgIIa*. As these 3 neuropeptidergic systems have been identified as stimulators of Lh in some teleost species (Popesku et al., 2008; Biran et al., 2012), this is suggestive of a type of compensation to maintain reproduction following multiple gene knockout. However, this does not fully explain the mechanism underlying a potential compensation. These changes are relatively minor and on the scale of a whole brain homogenate. Independent knockout of Gnrh and the kisspeptins does not change gonadotropin expression. Similarly, expression of *fshb* and *lhb*, and *gnih* and *gnrh2* did not change in either sex of the triple knockout line (Liu et al., 2017). I hypothesize that multiple neuronal systems in teleosts are functioning in parallel (Figure 1) to maintain appropriate patterns of Lh release. To completely disrupt this, 3 or more separate systems (i.e., Gnrh, kisspeptin plus a different neuropeptide or neurotransmitter represented by Nx in Figure 1) would have to be genetically modified.

## Concluding remarks

Experimentalists are attracted to fish for numerous reasons. These include their commercial importance and the ease of genetic modification to understand development or to model human diseases. Teleost fishes are also fascinating because they exhibit the greatest diversity of reproductive patterns among the vertebrates (Wootton and Smith, 2014). This ranges from the typical gonochoristic species with separate sexes, to the sex-changers and hermaphrodites. Currently, >33,000 fish species are recognized. This is close to 50% of all known vertebrate species. Of these, the Teleostei are the majority (~27,000 species). The main subjects of genetic modification (overexpression or knockout) to date have been but a few gonochoristic species.

Even within the few species investigated, significant diversity is evident. Differences in the progression of ovarian and testicular differentiation and of spawning tactics (daily versus annual) could be linked to different control mechanisms compared to mammals. It has been assumed by some investigators that teleosts and other non-mammalian vertebrates do not exhibit pulsatility in pituitary hormone section as observed in mammals, and that this may be the reason control mechanisms appear different. Yet, there is very good evidence to the contrary, so this cannot be the reason. Daily cycles of Lh secretion in goldfish represents low frequency diurnal activation of the hypothalamo-hypophysial axis (Hontela and Peter, 1978). Clear pulsatile Lh secretion in trout (Zohar, 1980; Zohar et al., 1986a,b) and chicken (Vizcarra et al., 2004) indicates that gonadotropin pulsatility is a common neuroendocrine feature. Fish also have a timed preovulatory Lh surge, like the tetrapods. Thus, it is probable that the multitude of interacting stimulatory and inhibitory inputs fine-tune basal secretion and drive pulses and surges of Lh in teleosts as they do in mammals. Alternatively, the multiplicity and differential appearance/disappearance of the 3 Gnrhs (or other peptides), through gene duplication and loss events, may have led to species-specific rewiring of neuroendocrine circuits as the teleosts diversified and adapted to new ecological niches. It is worthy to note that medaka (Order Beloniformes) and zebrafish (Order Cypriniformes) lineages diverged 115–200 Myr ago, providing ample time for genetic changes to occur (Signore et al., 2009).

Recent investigations have focused on the role of neuropeptides in teleost reproduction. This appears to be accompanied by reduced scientific interest in classical neurotransmitters. Dopamine is a major inhibitor of Lh in many species. *In vivo* and *in vitro* pharmacology has firmly established that glutamate, γ-aminobutyric acid, norepinephrine and serotonin have significant effects on hypothalamo-pituitary function in teleosts (Trudeau, 1997; Popesku et al., 2008; Zohar et al., 2010). Their essentiality for successful reproduction should also be explored through genetic manipulation of synthesis enzymes, receptors and membrane transporters. As with the multiplicity of neuropeptides, the study of non-peptidergic neuroendocrine systems will also be challenging. This will be worthwhile, however, since neurotransmitters are key regulators of neuropeptidergic systems controlling teleost reproduction (Trudeau et al., 2000; Hasebe and Oka, 2017; Song et al., 2017).

## Author contributions

The author confirms being the sole contributor of this work and approved it for publication.

### Conflict of interest statement

The author declares that the research was conducted in the absence of any commercial or financial relationships that could be construed as a potential conflict of interest.

## Footnotes

^1^*In this review the zebrafish gene/protein nomenclature is followed for convenience. See:* https://wiki.zfin.org/display/general/ZFIN+Zebrafish+Nomenclature+Conventions.
